# Predictive Value of Jugulo-omohyoid Lymph Nodes in Lateral Lymph Node Metastasis of Papillary Thyroid Cancer

**DOI:** 10.1186/s12902-024-01576-7

**Published:** 2024-05-21

**Authors:** Huizhu Cai, Lingdun Zhuge, Zehao Huang, Shixu Wang, Ping Shi, Dangui Yan, Minghui Wei, Lijuan Niu, Zhengjiang Li

**Affiliations:** 1 Department of Head and Neck Surgery, National Cancer Center/National Clinical Research Center for Cancer/Cancer Hospital, Chinese Academy of Medical Sciences and Peking Union Medical College, Beijing, China; 2 Department of ENT, The Fourth Affiliated Hospital of Hebei Medical University, Shijiazhuang, China; 3 Department of Head and Neck Surgery, National Cancer Center/Cancer Hospital & Shenzhen Hospital, Chinese Academy of Medical Sciences and Peking Union Medical College, Shenzhen, China; 4 Department of Ultrasound, National Cancer Center/National Clinical Research Center for Cancer/Cancer Hospital, Chinese Academy of Medical Sciences and Peking Union Medical College, Beijing, China

**Keywords:** Papillary thyroid carcinoma, Jugulo-omohyoid lymph nodes, Lymph node metastasis, Neck dissection, Predict model

## Abstract

**Background:**

Jugulo-omohyoid lymph nodes (JOHLN) metastasis has proven to be associated with lateral lymph node metastasis (LLNM). This study aimed to reveal the clinical features and evaluate the predictive value of JOHLN in PTC to guide the extent of surgery.

**Methods:**

A total of 550 patients pathologically diagnosed with PTC between October 2015 and January 2020, all of whom underwent thyroidectomy and lateral lymph node dissection, were included in this study.

**Results:**

Thyroiditis, tumor location, tumor size, extra-thyroidal extension, extra-nodal extension, central lymph node metastasis (CLNM), and LLMM were associated with JOHLN. Male, upper lobe tumor, multifocality, extra-nodal extension, CLNM, and JOHLN metastasis were independent risk factors from LLNM. A nomogram based on predictors performed well. Nerve invasion contributed the most to the prediction model, followed by JOHLN metastasis. The area under the curve (AUC) was 0.855, and the p-value of the Hosmer-Lemeshow goodness of fit test was 0.18. Decision curve analysis showed that the nomogram was clinically helpful.

**Conclusion:**

JOLHN metastasis could be a clinically sensitive predictor of further LLM. A high-performance nomogram was established, which can provide an individual risk assessment of LNM and guide treatment decisions for patients.

## Background

The incidence of thyroid cancer has increased rapidly worldwide in recent decades [1, 2]. Papillary thyroid carcinoma (PTC) is the most common type of thyroid malignancy, prone to early lymph node metastasis (LNM). PTC has an excellent prognosis, as a 20-year overall survival rate could reach 90%, and its disease-specific survival rate is approximately 97% [3]. Although it is acknowledged that LNM has a less significant factor influencing survival rate, several recent studies reported that LNM negatively affects long-term recurrence [4–8]. The most common LNM site of PTC is the central cervical lymph nodes, followed by lateral cervical lymph nodes.

According to the latest National Cancer Comprehensive Network (NCCN) guidelines (version 2023) and 2015 American Thyroid Association (ATA) guidelines, lateral lymph node dissection is only recommended for patients who have identified lateral lymph node metastasis (LLNM). Clinicians often judge the presence of LNM with preoperative imaging examination and intraoperative frozen pathology. However, the sensitivity of evaluations is limited [9], and some occult LNM might lead to relapse. Previous studies have shown that the incidence of occult LLNM in PTC patients could reach 18.6-64% [10–12].

Jugulo-omohyoid lymph nodes (JOHLN) are a group of lymph nodes that often lay on or behind the internal jugular vein and are fixed to the carotid sheath and intermediate tendon of the omohyoid muscle [13]. It has been reported that the frozen pathology of JOHLN appeared to be a valuable assessment for the clinically node-negative neck of oral carcinoma [14]. JOHLN may be also a potential predictor of LLNM of PTC, as it has been noticed that when injecting carbon nanoparticles into the thyroid during surgery, JOHLN was usually first traced [15].

Our previous study has proved that JOHLN was associated with an increased likelihood of LLNM [16]. This study aimed to assess the risk factor of JOHLN metastasis and explore the predictive value of JOHLN in LLNM to provide theoretical evidence for a reasonable decision of dissection extent in PTC patients.

## Materials and methods

### Patients

This retrospective study was conducted with clinical records collected at the Department of Head and Neck Surgical Oncology, Cancer Hospital of Chinese Academy of Medical Sciences and Peking Union Medical College from October 2015 to January 2020. Patients were enrolled if they met all of the following inclusion criteria: (i) patients had undergone primary thyroid lobectomy or total thyroidectomy by the same experienced clinician; (ii) patients had undergone ipsilateral or bilateral lateral lymph node dissection (LLND); (iii) patients were pathologically confirmed PTC; (iv) patients had no history of neck surgery or radioactive treatment; (v) patients had no history of other systematic malignant tumors. This study was approved by the ethics committee of the Cancer Hospital of Chinese Academy of Medical Sciences (reference number: NCC2016ST-23) and performed in accordance with the Declaration of Helsinki. Informed consent was obtained from all participants. After excluding patients with incomplete medical records, 550 patients were enrolled in this study.

### Surgery

We explained intraoperative and postoperative risks to all patients and their relatives in detail before surgery to ensure they fully understood the disease and surgical methods. Total thyroidectomy was performed on patients with bilateral PTC. For unilateral PTC patients, total thyroidectomy or lobectomy plus isthmusectomy were performed depending on patients’ wishes. Total thyroidectomy was recommended when unilateral PTC patients met one of the following conditions: tumor size > 4 cm, multifocality in one lobe, contralateral benign nodules, or distant metastasis (according to guidelines of Chinese Thyroid Association).

Central lymph node dissection was performed on all patients. Therapeutic LLND of level II-IV or II-V was performed on patients pathologically confirmed to have LLNM by fine-needle aspiration or intraoperative frozen biopsy in the lateral compartments. Meanwhile, we performed LLND of level III and IV based on the original thyroid collar incision after CLNM was confirmed by fine-needle aspiration or intraoperative frozen biopsy.

**Fig. 1:**
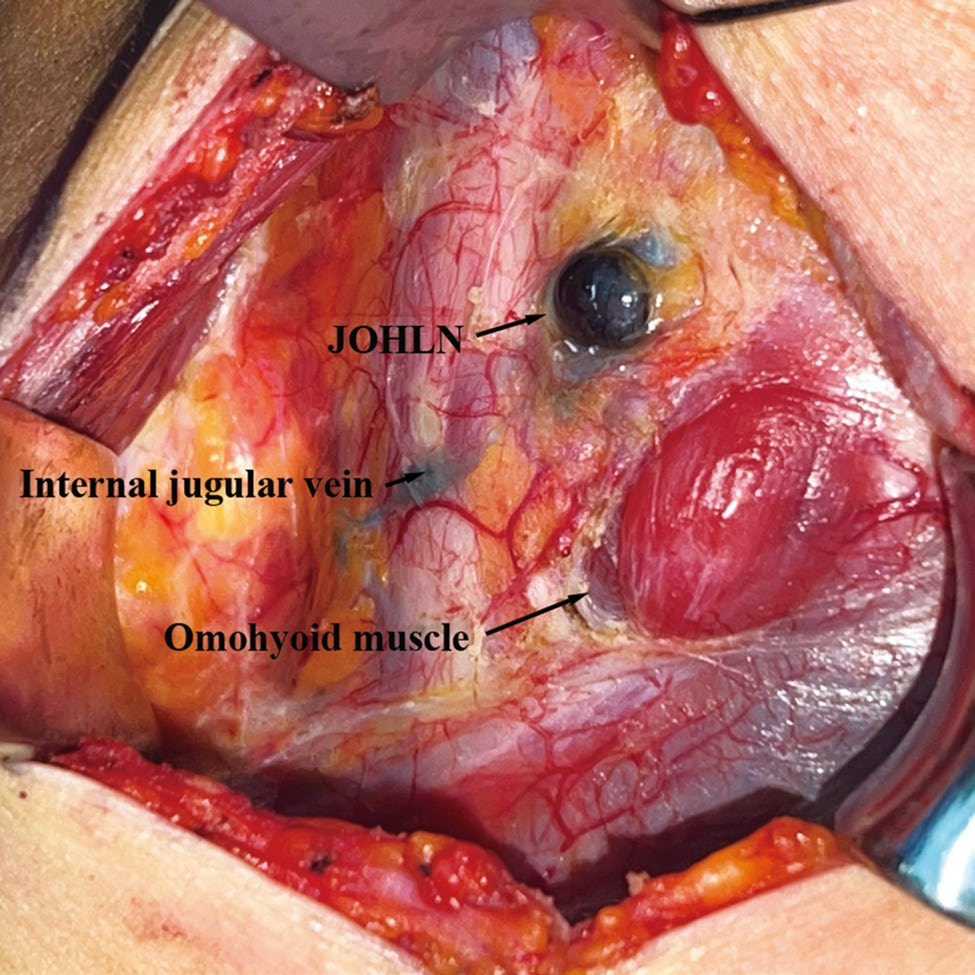
Intraoperative view of JOHLN after injecting tracing nanoparticle to thyroid gland

JOHLN were defined as the group of lymph nodes located in front of the internal jugular vein and at the intersection of the internal jugular vein and omohyoid muscle during surgery (Fig. 1). We routinely divided the specimens into JOHLN and posterior internal jugular vein lymph nodes for pathological examination.

### Statistical analysis

All statistical data were analyzed in SPSS version 26.0 (IBM Inc, Armonk, NY, USA) or R version 4.2.1(www.r-project.org). Categorial variables were expressed as numbers [percent (%)]and were compared by the chi-square test. *P* < 0.05 was considered statistically significant. Variables of *p* < 0.05 were included in multivariate logistic regression analysis. Forest plots were created to show a subgroup analysis of the association between LLNM and JOHLN. A nomogram was created by having all the variables with *p* < 0.1 in multivariate analysis to calculate individual probabilities. The nomogram model was calibrated using a calibration plot, and the Hosmer-Lemeshow goodness of fit test and the receiver operating characteristic (ROC) curves were used to test its discrimination. The clinical usefulness of the nomogram was evaluated using the decision curve analysis (DCA).

## Results

### Clinical features and pathological factors associated with JOHLN

Of all the 550 patients included, 176 patients (32.0%) were found to have JOHLN metastasis. The mean number of metastasized JOHLNs was 0.45 (range:0–6). LNM in central and lateral compartments was observed in 428(77.8%) and 421(76.5%) patients.

The clinicopathological characteristics of patients in the JOHLN + group and JOHLN- group are shown in Table 1. No statistically significant difference between the two groups was observed in gender distribution and age at surgery. Thyroiditis was less common in the JOHLN + group (*p* = 0.029). Metastatic JOHLN was related to upper lobe tumor (*p* = 0.006). The proportions of papillary thyroid microcarcinoma differed between patients with JOHLN metastasis and patients without JOHLN metastasis (*p* = 0.016). Patients in the JOHLN + group were more likely to exhibit extra-thyroidal extension and extra-nodal extension (*p* = 0.007 and *p* = 0.018). Regarding LNM, the JOHLN + group showed a higher incidence of central lymph node metastasis (CLNM) and LLNM than the JOHLN- group (*p* = 0.002 and *p* < 0.001). Furthermore, when analyzing separately, JOHLN metastasis was associated with metastasis of level II, III and IV (*p* < 0.001, *p* = 0.010, *p* < 0.001, respectively).

**Table 1:** Clinical and pathological characteristics of JOHLN + and JOHLN- patients

Clinicopathologic	JOHLN metastasis		Univariate analysis	Multivariate analysis	
variables	Present(*n* = 176)	Absent(*n* = 374)	p value	OR(95% CI)	p value
Male	61(34.7%)	112(29.9%)	0.267		
Age ≥ 55years	18(10.2%)	51(13.6%)	0.260		
Thyroditis	12(6.8%)	49(13.1%)	**0.029**	0.495(0.251–0.979)	**0.043**
Upper lobe	61(34.7%)	88(23.5%)	**0.006**	1.780(1.174–2.697)	**0.007**
Tumor size ≥ 10 mm	132(75.0%)	242(64.7%)	**0.016**	1.359(0.882–2.902)	0.164
Extra-thyroidal extension	149(84.7%)	278(74.3%)	**0.007**	1.349(0.814–2.236)	0.245
Extra-nodal extension	67(38.1%)	105(28.1%)	**0.018**	1.120(0.746–1.681)	0.585
CLNM	151(85.8%)	277(74.1%)	**0.002**	1.577(0.924–2.692)	**0.095**
LLNM	156(88.6%)	265(70.9%)	**< 0.001**	2.449(1.411–4.253)	**0.001**
Level II LNM	147(83.5%)	206(55.1%)	**< 0.001**		
Level III LNM	116(65.9%)	203(54.3%)	**0.010**		
Level IV LNM	123(69.9%)	199(53.2%)	**< 0.001**		

JOHLN Jugulo-omohyoid lymph nodes, CLNM central lymph node metastasis, LLNM lateral lymph node metastasis

When the multivariate analysis was performed, statistically significant differences were found in thyroiditis (absent, *p* = 0.043), location of the tumor (upper lobe, *p* = 0.007), CLNM (*p* = 0.095), and LLMM (*p* = 0.001). However, no statistically significant difference was observed for tumor size, extra-thyroidal extension, and extra-nodal extension, as shown in Table 1.

**Fig. 2:**
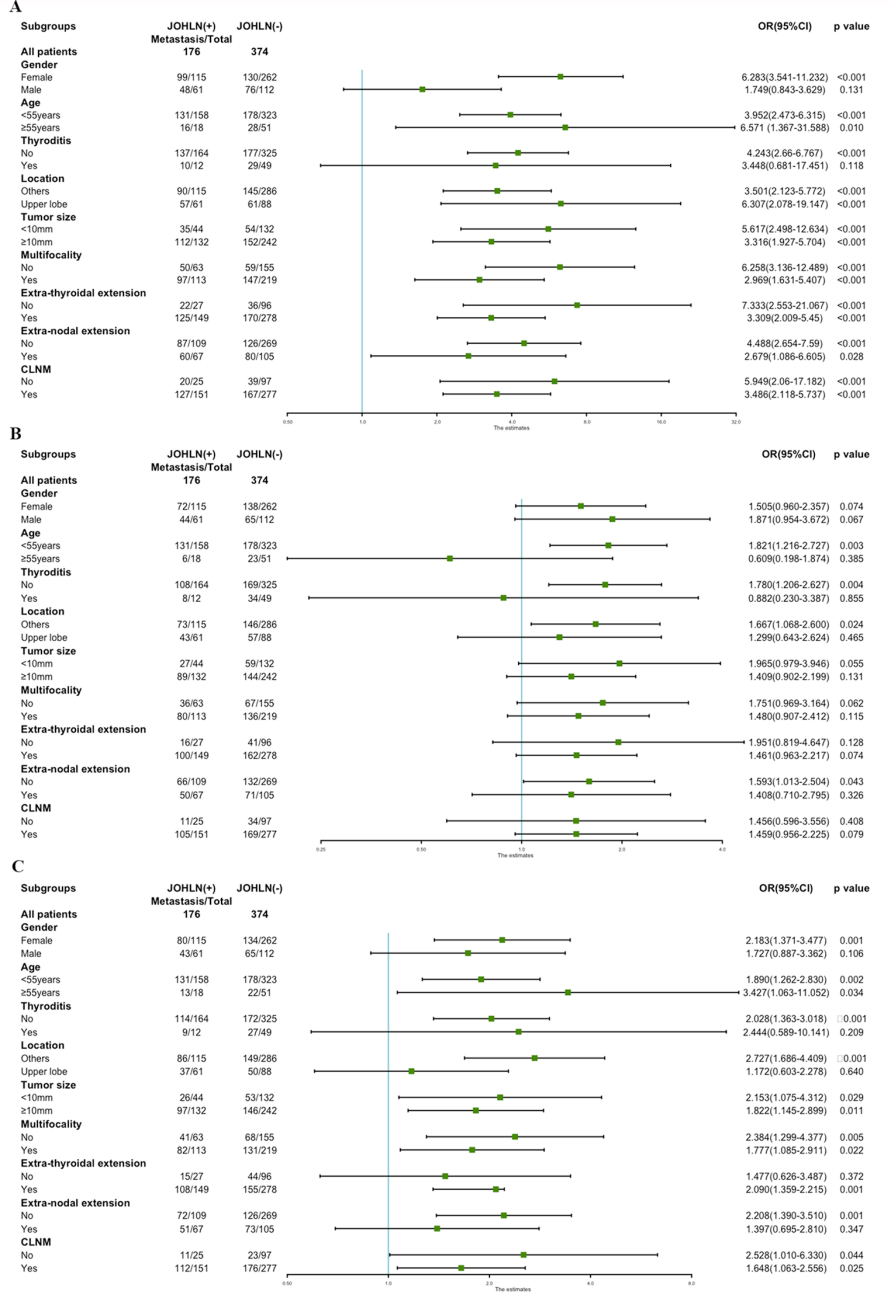
Subgroup analysis of association between lateral lymph node metastasis and JOHLN metastasis according to baseline characteristics. (**A**) Association between level II LNM and JOHLN metastasis. (**B**) Association between level III LNM and JOHLN metastasis. (**C**) Association between level IV LNM and JOHLN metastasis

Furthermore, the stratified analysis revealed that JOHLN metastasis was statistically and independently correlated with level II, III and IV LNM in most subgroups (Fig. 2).

### Correlation between LLNM and JOHLN

When cases with LLNM were compared to patients without, univariate analyses showed a statistically significant difference in the following variables: male (*p* = 0.006), age ≥ 55 years(*p* = 0.038), upper lobe tumor(*p* = 0.072), tumor size ≥ 10 mm(*p* < 0.001), multifocality(*p* < 0.001), nerve invasion(*p* = 0.013), extra-thyroidal extension(*p* = 0.001), extra-nodal extension(*p* < 0.001), CLNM(*p* < 0.001) and JOHLN metastasis(*p* < 0.001), as shown in Table 2.

**Table 2:** Univariate and multivariate analyses of LLNM in PTC

Variables	LLNM		Univariate analysis	Multivariate analysis	
	Present(*n* = 421)	Absent(*n* = 129)	p value	OR(95% CI)	p value
Male	145(34.4%)	28(21.7%)	**0.006**	1.706(1.009–2.884)	**0.046**
Age ≥ 55years	46(10.9%)	23(17.8%)	**0.038**	0.757(0.394–1.453)	0.402
Thyroditis	28(11.4%)	13(10.1%)	0.675		
Upper lobe	122(29.0%)	27(20.9%)	**0.072**	1.899(1.086–3.322)	**0.025**
Tumor size ≥ 10 mm	304(72.2%)	70(54.3%)	**< 0.001**	1.510(0.932–2.446)	0.094
Multifocality	278(66.0%)	54(41.9%)	**< 0.001**	2.687(1.692–4.268)	**< 0.001**
Nerve invasion	26(6.2%)	1(0.8%)	**0.013**	7.915(0.950–65.930)	0.056
Extra-thyroidal extension	341(81.0%)	86(66.7%)	**0.001**	1.149(0.682–1.937)	0.602
Extra-nodal extension	157(37.3%)	15(11.6%)	**< 0.001**	2.457(1.319–4.576)	**0.005**
CLNM	360(85.5%)	68(52.7%)	**< 0.001**	4.461(2.654–7.499)	**< 0.001**
JOHLN(+)	156(37.1%)	20(15.5%)	**< 0.001**	2.479(1.407–4.368)	**0.002**

LLNM lateral lymph node metastasis, CLNM central lymph node metastasis, JOHLN Jugulo-omohyoid lymph nodes

The multivariate analysis revealed that male(*p* = 0.046), upper lobe tumor(*p* = 0.025), multifocality(*p* < 0.001), extra-nodal extension(*p* = 0.005), CLNM(*p* < 0.001) and JOHLN metastasis(*p* = 0.002) were independent risk factors from LLNM (Table 2).

### Diagnostic model development

To better determine the ability of JOHLN metastasis to predict LLNM, we excluded those patients who did not identify JOHLN. A total of 329 patients were included in the following analyses. For lateral compartment metastasis, JOHLN metastasis had respective sensitivity, specificity, positive and negative predictive values of 67%, 79%, 89%, and 50%. For metastasis of level II, JOHLN status had sensitivity, specificity, and positive and negative predictive values of 75%, 78%, 84%, and 67%, respectively. (Table 3).

**Table 3:** Ability of JOHLN metastasis to predict LLN

Lymph node	Sensitivity	Specificity	PPV	NPV	LR+	LR-
metastasis	(%)	(%)	(%)	(%)		
Lateral	67	79	89	50	3.21	0.42
Level II	75	78	84	67	3.40	0.32
Level III	69	63	66	66	1.85	0.49
Level IV	69	65	70	63	1.94	0.48

PPV positive predictive value, NPV negative predictive value, LR + positive likelihood radio, LR- negative likelihood ratio

**Fig. 3:**
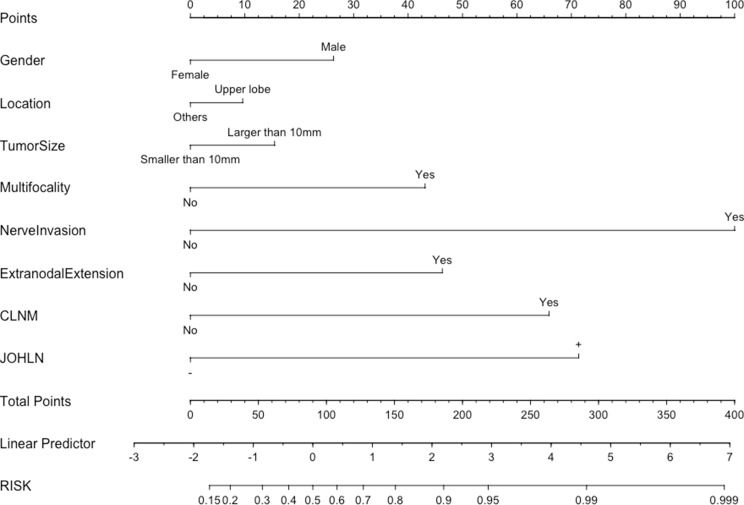
Risk factor-based nomogram for predicting LLNM. Nomogram instructions: to obtain the nomogram-predicted risk of LLNM, locate the patient values on each axis. Draw a vertical line to the points axis to determine how many points are attributable to each variable. Sum the points for all the variables. Locate the sum on the total points line to assess the patient’s predicted risk of LLNM

A nomogram was developed to calculate the degree of individual risk to improve the clinical diagnosis of LLNM. Eight risk factors, which were risk factors of *p* < 0.1 of LLNM as mentioned above, were included in the nomogram (Fig. 3). Nerve invasion contributed the most to the prediction model, followed by JOHLN metastasis. When all these criteria were satisfied, the specificity was nearly 100%.

**Fig. 4:**
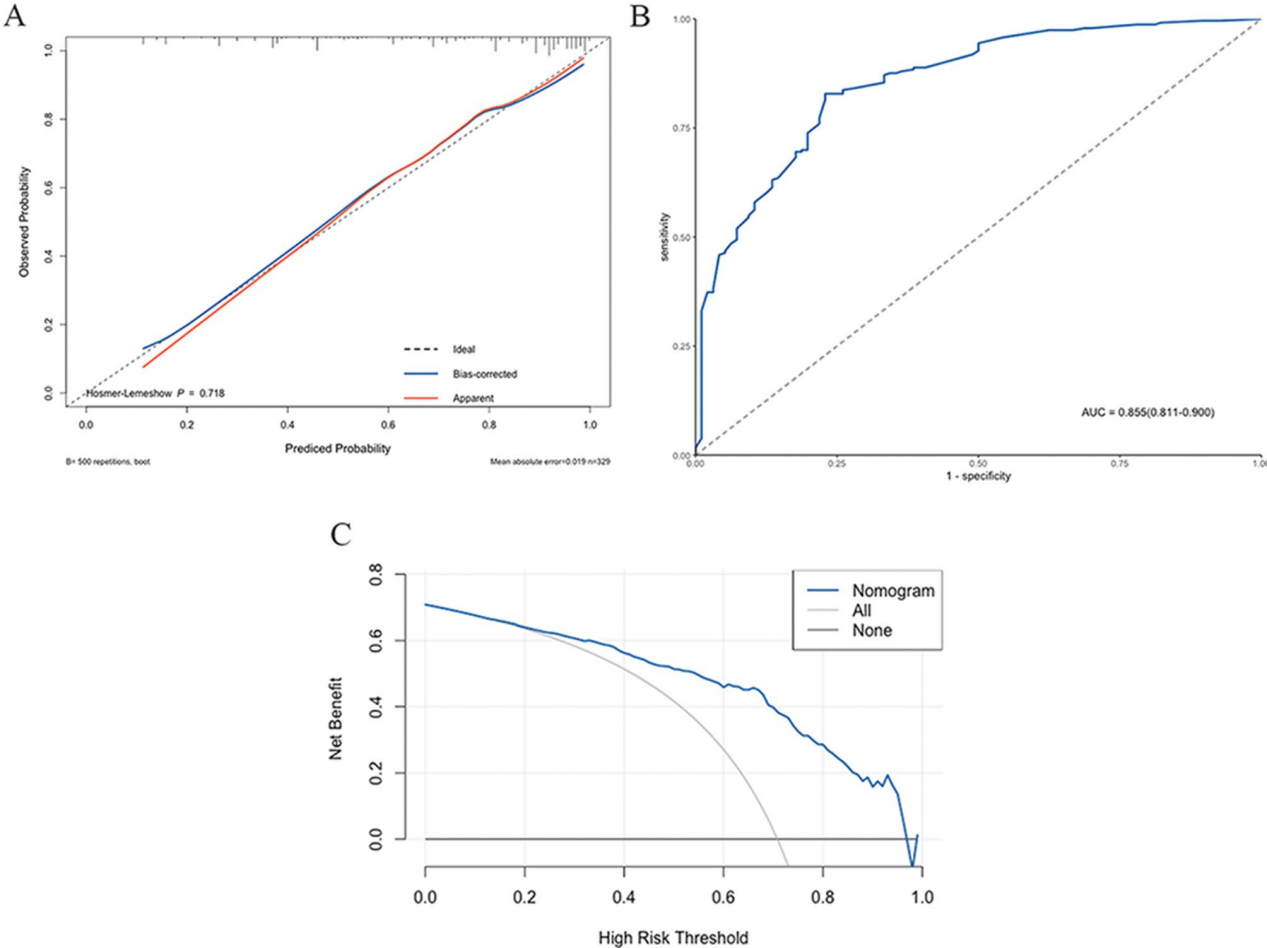
The efficiency of the nomogram for predicting LLNM evaluated by (**A**) calibration curves, (**B**) the ROC curve, (**C**) decision curve

The internal calibration plot showed a mostly perfect agreement between the predicted and actual results of the nomogram model, as shown in Fig. 4A. The Hosmer-Lemeshow goodness of fit test also demonstrated an excellent concordance between the predicted and actual outcomes (*p* = 0.718). As the ROC curve was shown in Fig. 4B, the area under curve (AUC) was 0.855, which indicated good discrimination of the model. In the DCA curve, when the threshold probability was more than 0.130, the nomogram model achieved a more significant net benefit than the “None” or “All” (Fig. 4C).

## Discussion

It is generally believed that the cervical lymph node metastasis of PTC disseminates sequentially to the central compartment and later to the lateral neck. However, the lymphatic drainage of the thyroid is more complex than we thought. Several studies have reported that even each lobe of the thyroid gland has its internal lymphatic system, and there is also communication between multiple drainages around the gland [17–19]. The rate of skip metastasis, which means LLNM occurs without the involvement of the central compartment, in PTC ranges from 1.6 to 21.8% [17, 20].

Although there are many arguments about the exact extent of LND for PTC patients, current consensus and guidelines do not recommend prophylactic LLND for patients with unidentified LLNM. However, some authors have found that the incidence of occult LLNM in PTC patients was higher than we thought [10–12]. This may be caused by limitations of preoperative examinations, such as ultrasound and CT scan, which play an essential role in determining the surgical field. There has been no clear consensus on whether occult metastasis will increase the risk of recurrence or cause a worse prognosis, but a study reported that higher lymph node yield from dissection was associated with a lower rate of recurrence [21].

For all the reasons above, we urgently need a predictive model to determine potential LLNM and minimize the possibility of under-treatment and incidence of recurrence. In this study, we assessed the predictive value of JOHLN in LLNM to explore a reasonable prediction model of LLNM to benefit clinical practice. JOHLN is a group of lymph nodes in level III, and because of its shortest drainage distance, it is usually the first dissemination of the lateral neck from the thyroid. We notice this from injecting carbon nanoparticles, a kind of approval lymphatic tracer, into the thyroid during surgery. JOHLN is usually first traced in the lateral neck within a few minutes [15]. This makes JOHLN a potential predictor of LLNM of PTC. Our study showed a similar result when calculating the risk factors of JOHLN metastasis. In our series, thyroiditis, upper lobe tumors, larger tumor size, extra-thyroidal extension, extra-nodal extension, and CLNM were related to JOHLN metastasis. Tumor location and CLNM were independent risk factors of JOHLN metastasis, while thyroiditis was a protective factor. This was similar to the results of previous studies that CLNM, extra-thyroidal extension, male sex, upper pole location, and tumor size were risk factors of LLNM, and thyroiditis reduced risk meanwhile [22, 23]. We also noticed that JOHLN metastasis was not only related to LLNM but also correlated with level II, III and IV LNM in most subgroups.

Apart from the correlation between JOHLN and LLNM, when analyzing the relationship between LLNM and JOHLN, we found that there was a significant correlation. The sensitivity, specificity, positive and negative predictive values of JOHLN for predicting LLNM were respective 67%, 79%, 89% and 50%. And significantly, when only predicting LNM of level II, the sensitivity, specificity, positive and negative predictive values rose to 75%, 78%, 84% and 67%, respectively. We know that the occurrence probability of LNM in the lateral neck is: level III > level IV > level II > level V [24]. Since JOHLN is part of level III, it suggested that JOHLN may be the channel for the thyroid tumor to metastasize to level II. By clarifying the metastasis of JOHLN before or during the operation, we can determine the extent of surgery and avoid occult metastasis.

A predictive nomogram was constructed based on the significant factors related to LLNM. As far as we can see, there is no report on using a monogram including JOHLN metastasis to predict LLNM. The calibration plot showed good agreement between the predicted and actual probability of LLNM. Moreover, the AUC of the nomogram was 0.855, which was much greater than the value of superior accuracy of 0.7. And DCA curve showed that most PTC patients could benefit from our nomogram. Thus, this nomogram could aid clinicians in deciding whether to perform LLND rather than only deciding surgical extent based on preoperative ultrasound and intraoperative frozen pathology examination.

Our nomogram showed that nerve invasion contributed the most to predicting LLNM, followed by JOHLN metastasis and CLNM. As perineurial invasion is usually diagnosed by postoperative pathology, it is hard to diagnose nerve invasion before or during surgery. This makes JOHLN metastasis and CLNM, which could be determined by intraoperative frozen pathology, the most valuable predictor of LLNM. Combined with our experience, we first take lymph nodes in level VI and JOHLN for frozen pathological examination when further LNM is suspected in examinations before surgery based on the original thyroid collar incision, and then complete thyroidectomy. If metastasis is found, the incision is extended along the skin striae to the posterior edge of the sternocleidomastoid muscle to complete the LLND. This would not prolong surgery time in waiting for frozen pathology, but also avoid the possibility of omission.

## Conclusion

JOLHN metastasis is associated with several adverse prognostic factors, including LLNM. As JOLHN could be a clinically sensitive predictor of further LLM, especially the lateral compartment, we develop a predictive model that includes all factors related to LLNM. Intraoperative frozen pathology of JOHLN is strongly recommended, and if LLNM were to be found, appropriate LND should be performed to achieve a favorable low recurrence rate.

## Data Availability

The data that support the findings of this study are available on request from corresponding authors.
